# Cryoballoon Ablation for Atrial Fibrillation

**DOI:** 10.1016/s0972-6292(16)30479-x

**Published:** 2012-04-30

**Authors:** Jason G Andrade, Marc Dubuc, Peter G Guerra, Laurent Macle, Lena Rivard, Denis Roy, Mario Talajic, Bernard Thibault, Paul Khairy

**Affiliations:** Electrophysiology Service, Department of Cardiology, Montreal Heart Institute, Universite de Montreal, Montreal, Canada

**Keywords:** Cryoballoon Ablation, Atrial Fibrillation

## Abstract

Focal point-by-point radiofrequency catheter ablation has shown considerable success in the treatment of paroxysmal atrial fibrillation. However, it is not without limitations. Recent clinical and preclinical studies have demonstrated that cryothermal ablation using a balloon catheter (Artic Front©, Medtronic CryoCath LP) provides an effective alternative strategy to treating atrial fibrillation. The objective of this article is to review efficacy and safety data surrounding cryoballoon ablation for paroxysmal and persistent atrial fibrillation. In addition, a practical step-by-step approach to cryoballoon ablation is presented, while highlighting relevant literature regarding: 1) the rationale for adjunctive imaging, 2) selection of an appropriate cryoballoon size, 3) predictors of efficacy, 4) advanced trouble-shooting techniques, and 5) strategies to reduce procedural complications, such as phrenic nerve palsy.

## Introduction

Atrial fibrillation (AF) is the most common sustained cardiac arrhythmia. It accounts for the majority of arrhythmia-related emergency room visits and hospital admissions,[1-3] and is associated with reductions in quality of life, functional status, cardiac performance, and overall survival.[1] Catheter ablation, which is centered on electrical isolation of triggering foci within the pulmonary veins (PV) through circumferential lesions around PV ostia, has been shown to result in sustained improvements in quality of life, decreased hospitalizations and, potentially, improved survival.[4-6]

Radiofrequency (RF) catheter ablation has shown considerable success in treating symptomatic AF, particularly in comparison to anti-arrhythmic drugs [7,8]. Unfortunately, major complications including thromboembolism, cardiac perforation, and injury to adjacent structures are not infrequently observed [2,3,7,9,10]. Further, the procedure is complex, time-consuming and highly dependent on operator competency given the inherent difficulties associated with creating contiguous curvilinear lesions with a technique originally developed for focal ablation. As such, considerable effort has been directed towards developing technologies specifically for PV isolation (PVI) as a means to achieve safer and more effective PVI that is less reliant on operator dexterity. Recently clinical and preclinical studies have demonstrated that cryothermal ablation using a balloon catheter (Artic Front©, Medtronic CryoCath LP, Kirkland, Canada) is an effective alternative treatment for AF.

## Efficacy of Cryoballoon Ablation

To date over 20,000 cryoballoon-based PV ablation procedures have been performed worldwide. In a recent systematic review and meta-analysis, we reported the cumulative early experience with cryoballoon-based ablation (CBA).[11] CBA resulted in a high procedural success rate (>98% of patients achieving complete PVI) and 1-year freedom from recurrent AF (single cryoballoon procedure off anti-arrhythmic drugs 1-year success of 60%; 73% if a 3-month blacking period was included) [11,12]. In comparison, the longer-term freedom from recurrent AF after RF catheter ablation has been reported to be 50% to 64% after a mean follow-up of 14 months in the meta-analysis by Calkins et al. and 39.8±5.1% at 1 year in the prospective long-term cohort study by Weerasooriya et al.[7,13] Thus, the early experience suggests that cryoballoon ablation is efficacious for the maintenance of sinus rhythm at 1 year in patients with paroxysmal AF.

When compared to other rhythm control strategies, CBA has performed favourably. (See Table 1) The first randomized trial comparing AAD therapy and cryoballoon ablation, the Sustained Treatment of Paroxysmal Atrial Fibrillation (STOP-AF) trial, enrolled 245 patients with paroxysmal AF and randomized them (2:1) to cryoballoon-based PV isolation (n=163) or to AAD therapy (n=82).[14] The mean age of participants was 57 years, and those enrolled had already failed an average of 1.2 AADs. Balloon-only PVI was realised in 90.8% of participants, with an overall procedural success (≥3 PVs isolated) of 98.2% when focal cryoablation was added. Nineteen percent of patients required a repeat procedure within the 3-month blanking period. At 12 months of follow-up, 69.9% of the cryoballoon group (114/163) vs. 7.3% of the AAD group (6/82) were free of recurrent AF (p<0.001). Moreover, there was a statistically significant improvement in symptoms and quality of life in the cryoablation group. For all quality-of-life metrics, the improvement was greater in the cryoballoon group when compared to the AAD group.

Likewise, in comparison to other contemporary AF ablation technologies, CBA has performed favourably. In general, CBA is associated with procedure and fluoroscopy times that are somewhat longer than duty-cycled multi-electrode RF ablation but shorter than conventional RF ablation.[15-19] Despite these procedural differences, efficacy outcomes at all of time points sampled did not differ between CBA and conventional, magnetic guided, and duty-cycled multi-electrode RF ablation. [15-19,20]

## Safety of Cryoballoon Ablation

Major complications have been reported in approximately 5-6% of patients undergoing RF ablation for AF [7,9,10]. The rate of acute procedural complications reported with cryoballoon-based ablation (CBA) is relatively low (<3-5%) and compares favourably with irrigated RF and duty-cycled multi-electrode RF ablation [7,9,10,21]. With CBA, the reported rate of peri-procedural stroke or transient ischemic attack (TIA) is 0.3%, cardiac tamponade 0.6%, and groin complications 1.8%. In comparison, corresponding reported complication rates with RF ablation are 0.3-0.9% for stroke or TIA, 0.8-1.3% for cardiac tamponade, and 1-1.5% for groin complications.[11] Longer-term complications such as symptomatic PV stenosis and esophageal injury occurred infrequently with CBA (0.17% for symptomatic PV stenosis and a 0% incidence of atrial-oesophageal fistula).[11] Given the inclusion of patients early in the operators' learning curves, it is possible that the rate of complications with CBA will decrease further as operators gain experience.

While the global complication rate with CBA appears comparable to conventional RF ablation, three complications merit further discussion. Initially described in the early animal studies by Sarabanda et al., phrenic nerve palsy (PNP) has proven to be the most frequently observed complication with CBA, occurring in approximately 6% of clinical procedures (range 3-11%) [11,22,23]. While PNP can be observed after AF ablation regardless of the energy source, it occurs disproportionately more frequently with balloon-based ablation technologies when compared to conventional RF ablation [7,9,10]. Fortunately, despite PNP being a relatively common occurrence, the majority of cases are transient with <0.4% of PNPs persisting greater than one year.[11] However, while persistent PNP is rare, the incidence appears to be approximately twice that reported with conventional RF ablation (0.17%).[9]

The most commonly employed preventative measure is the use of continuous abdominal palpation during phrenic nerve pacing from a catheter placed in the superior vena cava cranial to the right-sided PVs. However, despite early interruption of ablation with the perceived onset of less vigorous diaphragmatic contractions, PNP continues to be observed. As such, efforts have been directed towards determining a more effective method for detecting early changes to the phrenic nerve. One such approach involving the use of diaphragmatic electromyography (EMG) has recently been published.[22] During phrenic nerve pacing, a reproducible supramaximal diaphragmatic compound motor action potential (CMAP) can be reliably recorded, providing valuable information about phrenic nerve function. In a recent animal study, it was demonstrated that a 30% reduction in CMAP amplitude reliably predicted impending hemidiaphragmatic paralysis (presaging diaphragmatic paralysis by abdominal palpation by 31 ± 23 seconds).[22] The first clinical application of this technique has also been reported.[24]

The second complication that warrants a more in depth discussion is that of PV stenosis, as its incidence with CBA is somewhat controversial. Prior to the recent STOP-AF trial, PV stenosis was thought to be non-existent after CBA. While multiple studies employing the use of systematic screening reported no PV stenosis (0/550 patients), the landmark STOP-AF trial noted a 3.07% (7/228 patients) incidence of radiographic PV stenosis [14,25-3214,25-32]. It is most likely that this incongruity reflects differences in criteria to define significant PV stenosis. In the AF ablation literature, PV stenosis is most commonly defined on the basis of diameter measurements (typically as a reduction in PV diameter <70%).[8] In contrast, STOP-AF defined PV stenosis as a <75% reduction in cross-sectional area from baseline, which corresponds to a 50% reduction in PV diameter.[14] As such, this more liberal definition may represent a relative overestimate of the rate of PV stenosis and limits direct comparisons to other studies. Even so, when the results of STOP-AF were combined with other studies employing systematic screening with non-invasive imaging, the incidence of radiographic PV stenosis was 0.90% (7/773 procedures), which is approximately half the rate observed in a large meta-analysis of conventional AF ablation with RF [7,25,32]. Reassuringly, the rate of symptomatic PV stenosis or PV stenosis requiring intervention was low (0.17%) and comparable to that observed with RF (0.1-0.3%) [7,9,10].

The final complication that warrants a detailed discussion is that of systemic thromboembolism. Cerebral ischemic events are a recognized complication of left atrial catheter ablation. In the recent systematic review of CBA, the incidence of thromboembolic complications, including peri-procedural stroke or TIA, was 0.32%, which compares favourably to conventional RF ablation (0.3-0.94%) [7,9,10].

Recognising that not all embolic cerebral events lead to clinical symptomatology, a series of studies recently examined signs of subclinical disease in an attempt to further delineate the risk of systemic thromboembolism after AF ablation. Sauren et al. examined the incidence of cerebral microembolic signals (MES) as a surrogate for neurological impairment and stroke in 30 patients undergoing percutaneous endocardial AF ablation: 10 patients undergoing segmental PVI using a 4-mm conventional (non-irrigated) RF ablation catheter, 10 patients undergoing segmental PVI using a 4-mm irrigated-tip RF catheter, and 10 patients undergoing circumferential PVI with CBA.[33] In this small study, the authors demonstrated a significantly lower incidence of MES in the middle cerebral arteries with CBA and irrigated RF catheters when compared to conventional non-irrigated RF (935±463 and 1,404±981 versus 3,908±2816 total MES respectively). Similarly, two recent studies comprising a total of 182 patients compared the incidence of silent cerebral ischemic lesions after PV isolation with duty-cycled multi-electrode ablation, conventional irrigated-tip RF ablation, and CBA. In both studies the incidence of new silent cerebral ischemic lesions, which have been associated with neurocognitive decline, was found to be significantly higher with multi-electrode ablation (37.5-38.9%) when compared to irrigated RF (7.4-8.3%) or CBA (4.3-5.6; overall P=0.008 for Siklody et al, and overall P=0.002 for Gaita et al.) [34,35].

## How to Perform Cryoballoon Ablation

### Patient Selection

In current practice, cryoballoon-based AF ablation is reserved for patients undergoing their first left atrial ablation for drug-refractory symptomatic paroxysmal AF or early persistent AF. While over 20,000 cryoballoon procedures have been performed worldwide, the vast majority of patients undergoing the procedure have had paroxysmal AF.

### Left Atrial Access

The ablation procedure is commenced by gaining left atrial access using a modified Brockenbrough technique and standard transseptal sheath [typically SL0, SL1 or SR0]. It is important to ensure that transseptal puncture used for cryoballoon ablation is located in a low anterior position within the membranous septum. Punctures in a more posterior position or within the muscular septum limit the manoeuvrability of the cryoballoon-FlexCath apparatus, potentially leading to technical challenges.

Early in our experience, we evaluated PV potentials (PVPs) though the introduction of a conventional diagnostic spiral catheter through a second more posteriorly located transseptal puncture. Recently, we modified our technique to perform only a single transseptal puncture. Evaluation of PVPs is now performed through the use of a small calibre 15 or 20-mm diameter circular mapping catheter introduced into the central lumen of the cryoballoon catheter (Achieve catheter; Medtronic; Minneapolis, MN). The Achieve catheter serves the dual purpose of acting as guide wire, as well as providing real-time verification of PVI during the freezing cycle, a period during which the placement of a conventional diagnostic spiral catheter is precluded by cryoballoon-induced PV occlusion. A third possible approach, one that we do not advocate due to additional risks that we consider unnecessary, is to perform a single transseptal puncture and serially exchange the cryoballoon catheter for a conventional spiral catheter. It should be noted that none of these approaches have been demonstrated to positively or negatively effect long-term outcomes and, as such, the decision to perform 1 or 2 transseptal punctures is based on physician preference.

Once left atrial access is obtained, a heparin bolus and subsequent infusion are commenced to target activated clotting times of 300 seconds. At the operator's discretion, selective or non-selective pulmonary venography is performed in the antero-posterior projection using a pigtail, multipurpose (MP), or National Institute of Health (NIH) catheter to delineate PV anatomy. Thereafter, a 300-cm 0.035-inch J-tipped guide wire is positioned in the left superior PV and the standard transseptal sheath is exchanged over the wire for a 12-Fr deflectable delivery sheath (15-Fr outer diameter; FlexCath, Medtronic, Minneapolis, MN).

### The Use Of Adjunctive Imaging

The use of adjunctive pre-procedural and intra-procedural imaging has remained a matter of physician preference. Although contentious, there are some reports suggesting that atypical PV anatomy such as common PV ostia may influence longer-term efficacy [18,36]. Kubala et al. evaluated PV anatomic patterns in 118 consecutive patients with drug refractory paroxysmal (72%) or persistent (28%) AF [36]. The authors demonstrated that the presence of a normal PV pattern is associated with fewer recurrences of AF at 13 months of follow-up, when compared to patients with left common ostia (67% vs. 50%, P = 0.02). The authors postulated that this difference might represent increased difficulty in obtaining circumferential lesions in the common ostia due to larger ostial diameters, an atypical ostial circumferential geometry, and/or a larger border zone between PV tissue and atrial myocardium in comparison to non-common PV ostia. In contrast, Sorgente et al. found no difference in the degree of PV occlusion or longer-term efficacy endpoints between patients with a left common ostium and those with normal branching of left PVs [18]. Given the conflicting reports regarding the influence of atypical PV anatomy on longer-term efficacy outcomes, we do not consider the presence of common PV ostia to contraindicate CBA [18].

As such, in our opinion, the main benefit of adjunctive imaging lies in the assessment PV diameter and selection of the appropriately sized cryoballoon [26,37]. We rely on the combination of pre-procedural cardiac magnetic resonance imaging and intra-procedural pulmonary venography and/or intracardiac echocardiography to define pulmonary venous anatomy and calibre. While venography is widely available and easy to perform, the use of intraprocedural intracardiac or transesophageal echocardiography provides a supplemental assessment of PV occlusion through the use of colour flow doppler (CFD) imaging. Total occlusion of the PV, as confirmed by CFD, successfully predicts PVI with a positive predictive value of 93-98%. Conversely, a negative predictive value for successful PVI of 92-100% was found in cases of persistent leak [30,38]. While it has been reported that procedure and fluoroscopy times can be reduced through the use of intracardiac echocardiography, the use of pre- or intraprocedural imaging has not been shown to directly affect longer-term efficacy. As such, the use of pre-procedural or intraprocedural imaging are largely based upon cost, physician preference, and regional patterns of practice.

### Choosing a Cryoballoon: 23 mm vs. 28 mm

The current generation cryoballoon catheter is available in two sizes, i.e., with 23 or 28 mm diameter balloons. Some operators prefer exclusive use of the larger 28 mm cryoballoon, whereas others have adopted a "toolbox" approach, with selective use of both the 23 mm and 28 mm cryoballoon. Advocates for the single large cryoballoon approach highlight the high rate of PNP retrospectively observed with the 23 mm balloon, while proponents of the toolbox approach emphasize the potential for increased long-term efficacy [25,39].

The premise underlying the argument for exclusive use of the larger 28 mm cryoballoon relates to the relative cryoballoon to PV (CB-PV) diameter and how that relationship influences the position of the resultant lesion. Early attempts to explain the excess incidence of PNP in association with 23 mm cryoballoon ablation (12.4% vs. 3.5% incidence with the 28 mm cryoballoon) postulated that the deployment of relatively undersized balloons deep inside the PV could result in an increased risk of PNP [40]. In addition to minimising the physical distance between the cryoballoon and phrenic nerve, CBA at a relatively more distal site within the RSPV results in a local environment more conducive to enhanced "cold" transfer to deeper tissue due to less convective heating of the balloon by atrial blood flow. As such, the "freeze" affected by a more distal cryoballoon position can be expected to result in deeper penetration of cryoenergy. Consequently, some operators have suggested that the incidence of PNP can be minimized through the exclusive use of the 28-mm balloon [15,25,40]. However, re-examination of the evidence has cast doubt on the validity of this assertion. While the observed early excess of PNP with the 23 mm CBA cannot be denied, it is important to note that the exclusive use of larger balloons has not entirely eliminated this complication. "Over-sizing" the balloon within the RSPV may result in: 1) mechanical distortion of the RSPV orifice with an even greater shortening of the relative distance between the cryoballoon and phrenic nerve, and 2) physical impingement of the phrenic nerve with resultant palsy [23].

It may be reasonably argued that the critical factor in preventing PNP is the avoidance of distal CBA by insuring that the diameter of the cryoballoon exceeds the diameter of the vein. While Chun et al recommended a critical PV to cryoballoon ratio of 0.93 when using the 28 mm cryoballoon for septal PVs, the authors did not derive a comparable ratio for left-sided veins nor for the 23 mm balloon [40]. In this regard, recent studies by Vogt et al. and Nadji et al. have shown that the 23 mm cryoballoon can be safely used to treat PVs smaller than 18 to 20 mm, respectively. Taken together, it appears that a CB-PV ratio of 0.9 may be appropriate for the 28 mm cryoballoon and that a CB-PV ratio of 0.8-0.85 may be preferable for the 23 mm balloon [37,39].

Our approach is to perform pre- and/or intra-procedural imaging to guide cryoballoon catheter selection. In general, when the PV diameter is less than 20 mm, we favour use of a 23 mm balloon. With larger PV diameters or common ostia, we resort to the use of a 28 mm cryoballoon.

### Balloon Preparation and Ablation

Once the appropriate balloon is selected, the central lumen is flushed with saline prior to insertion of an extra support, 0.035-inch J-tipped guide wire (or Achieve catheter) via a Tuohy-Borst adaptor. Thereafter, the cryoballoon catheter is advanced through the FlexCath into the left atrium where the guidewire (or Achieve catheter) is advanced into the targeted PV. We begin by targeting left-sided veins, working superiorly to inferiorly. Once the targeted vein is engaged with the guidewire, the cryoballoon is inflated in the body of the left atrium and advanced over the wire to the PV ostium. It is important to first inflate the balloon in the left atrium rather than inside the PV in order to avoid distal inflation, which potentially increases the risk of PV damage, phrenic nerve injury, and pulmonary necrosis.

### PV Occlusion and Lasting Isolation

To achieve lasting PVI, circumferential tissue contact must be attained. Failure to fully occlude the targeted PV during CBA will result in convective heating by intervening blood, which reduces the efficiency of freezing and the durability of the lesion.

**Figure 1:**
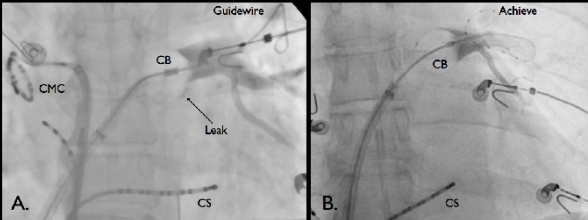
Cryoballoon Ablation of the Left Superior PV. Panel A. Cryoballoon (CB) ablation using a standard 0.035-inch J-tipped guidewire. A grade three occlusion is demonstrated with the persistence of a small inferior leak (Arrow). Axial catheter rotation and slight relaxation of the forward pressure resulted in complete occlusion (not depicted). Of note a circular mapping catheter (CMC) is pictured on the left side of the image in the right superior PV. Panel B. Cryoballoon ablation the Achieve mapping catheter. Complete (grade 4) occlusion is demonstrated. In both images a coronary sinus (CS) catheter is pictured.

Typically, the assessment of balloon occlusion is performed through the injection of 50% diluted contrast through the cryoballoon catheter's central lumen. (See Figure 1) In addition to providing confirmation of circumferential contact, which can be graded on a semi-quantitative scale from 1 (negligible occlusion with immediate rapid outflow from the PV) to 4 (total occlusion with complete contrast retention), contrast injection also provides information on the exact position of the inflated balloon in relation to the PV-LA junction. Alternative methods for assessing the adequacy of balloon occlusion include color flow doppler echocardiography (i.e. the absence of proximal flow on either transesophageal or intracardiac echo) as well pulmonary venous pressure assessment (i.e. the transition from a left atrial to pulmonary arterial pressure waveforms). The latter two methods allow for assessment of dynamic occlusion during cryoablation (i.e. with advanced manoeuvres such as the "pull down" technique), a period where confirmation of occlusion is not possible with contrast injection due to freezing of the central lumen of the cryoballoon catheter.

While every effort should be made to achieve optimal cryoballoon occlusion prior to ablation, it should be noted that a small degree of localized leak or delayed emptying of contrast (grade 3 occlusion) may be acceptable, since the onset of cryoablation is associated with balloon expansion, which may improve the seal.

### Management of Inadequate Seal

While left-sided veins and the right superior vein may usually be directly approached, variability in ostial geometry and orientation can impede adequate circumferential cryoballoon-PV contact. In this case, the simplest and most effective manoeuvre is the combination of axial catheter rotation, catheter flexion, and/or repositioning of the guidewire within a different distal PV branch in order to reorient the balloon's axis. It is important to note that the current generation cryoballoon catheter relies on four equatorial anteriorly directed refrigerant jets to produce the lowest ablation temperatures in a large circular zone from the balloon equator and extending a few mm towards its distal part. As such, should optimal occlusion only occur when the catheter shaft is misaligned compared to the axis of the PV, step-wise ablation should be performed from two different branches of the same vein to ensure that the entire circumferential surface of the PV ostium is adequately ablated.

In contrast to the superior veins, the inferior PVs present particular challenges due to the angulation of the transseptal sheath in relation to the PV ostia. In general, advancement of the cryoballoon catheter into the left atrium results in a cranial movement of the catheter. For the inferior veins, this results in a non-central alignment of the cryoballoon catheter in relation to the PV axis, favouring contact along the superior margin of the PVs at the expense of suboptimal contact inferiorly. Similar to the other PVs, direct engagement should initially be attempted. Should optimal occlusion not be obtained through the above-mentioned torsional or angular manoeuvres, use of more sophisticated techniques may be required.[41] In patients with an early branching inferior PV, the ***"hockey stick"*** technique can be used to optimize tissue contact along the inferior PV circumference. After engaging the early branching inferior PV with the guidewire, a maximal bend is applied to the sheath in the superior-posterior LA. The sheath is then advanced, allowing the balloon to be propelled into the inferior part of the PV ostium. In patients with no or only a late branching inferior PV, the ***"pull-down"*** technique can be used to optimize tissue contact along the inferior PV circumference. With this technique, the balloon is positioned parallel to the PV ostium to ensure optimal contact along the superior circumference of the PV. Cryoablation is then initiated regardless of the presence of an inferior leak. After approximately 60-90 seconds, when full N2O flow into the inner balloon is reached and the cryoballoon has adhered to the endocardium, the balloon and sheath are gently withdrawn or pulled down in order to seal the inferior margin of the PV. Of note, this manoeuvre must be performed with great care due to the potential risk of severe vascular damage associated with the application of excessive force. Lastly, in the case of an inferior RIPV ostium with a large persistent inferior leak precluding an effective "pull-down", the ***"big loop"*** technique can be used. For this technique, the sheath is maximally bent. While directed towards the lateral posterior LA, the guidewire is advanced along the posterior mitral annulus until the RIPV is reached. Thereafter, the sheath is positioned to guide the balloon over the wire into the RIPV ostium.

### Performing the Ablation Lesion and Intraprocedural Predictors of Success

When adequate circumferential contact is achieved, contrast material is flushed out of the catheter with saline, and a 240 second cryoablation is performed. Just prior to the onset of cryoablation, we apply gentle forward pressure on the cryoballoon catheter to maximise contact between the cryoballoon and PV antra.

Although trough-freezing temperatures below -80ºC are theoretically obtainable, in practice it is rare to achieve a trough freezing temperature below -60 to -70ºC. Cryoballoon temperatures are generally lower in the superior PVs when compared with inferior PVs, most likely due to passive warming of the proximal thermocouple by blood flow from the superior PVs [42]. A minimal cryoballoon temperature at the end of freezing of <-51ºC has been reported to predict PVI with 100% specificity for both superior and inferior PVs. In contrast, temperatures at 120 seconds of ≥ -36ºC for superior PVs and ≥ -33ºC for inferior PVs predicted ineffective PVI with 95% specificity (PPV 80%) and a 97% specificity (PPV 82%) respectively. As such, we interrupt cryoablation lesions that do not attain trough temperatures below -35ºC for inferior PVs and below -40ºC for superior PVs at 120 sec.

Similarly, whenever possible, we attempt to record real-time PV signals during ablation. A recent study by Dorwarth et al. demonstrated that sustained PVI was associated with a shorter mean time to conduction block (39 seconds) when compared to PVs with recovered PV conduction (125 seconds) [43]. The authors identified a cut-off time of 83 seconds for the prediction of stable sustained PVI without reconduction (86% sensitivity and 97% specificity). While this information is promising, we do not rely solely on this criterion to determine the utility (or lack thereof) for repeat lesions due to the lack of longer-term follow-up data. Moreover, in our experience with the Achieve catheter, real time recordings are obtainable in approximately 50% of PVs due to distal mapping catheter positioning (in order to achieve an adequate cryoballoon seal) or suboptimal circumferential mapping catheter-vessel wall contact.

### Phrenic Nerve Monitoring

**Figure 2:**
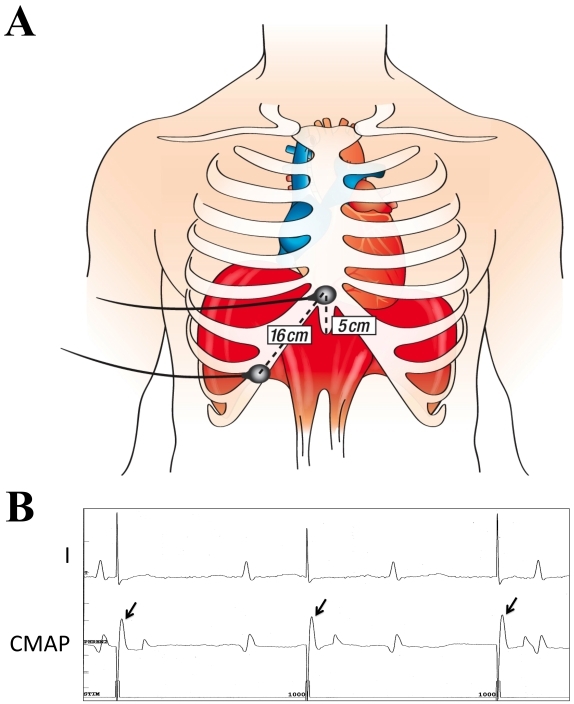
Configuration of surface electrodes (A) and corresponding recordings (B) of the diaphragmatic compound motor action potential (CMAP) . Panel A depicts the surface electrode configuration used to record the diaphragmatic compound motor action potential (CMAP). Electrodes are spaced 16 cm apart, one 5 cm above the xiphoid process and the second along the right costal margin. Shown in Panel B are surface tracings from lead I and from the thoracic leads positioned to record diaphragmatic CMAPs. The sweep speed is 100 mm/s. Note the stability of recorded CMAPs during right phrenic nerve pacing from the superior vena cava at 60 bpm. Reproduced with permission from Franceschi F, Dubuc M, Guerra PG, Khairy P. Phrenic nerve monitoring with diaphragmatic electromyography during cryoballoon ablation for atrial fibrillation: the first human application. Heart Rhythm. 2011;8(7):1068-1071. Copyright © Elsevier, 2011.

Prior to ablation of right-sided PVs, a standard 5-Fr quadripolar catheter is placed in the superior vena cava cranial to the right superior PV in order to pace the right phrenic nerve (10-15 mA at 1.0-2.0 msec pulse width at a cycle length of 1000 msec). In addition to direct palpation of the right hemi-diaphragmatic excursion, it is our practice to perform diaphragmatic electromyography (Figure 2). We immediately terminate cryoablation upon any perceived reduction in the strength of diaphragmatic contraction or a 30% reduction in the diaphragmatic CMAP amplitude. Of note, if the procedure is performed under general anaesthesia, it is essential to discontinue use of paralytic agents at least 30 minutes prior to planned phrenic nerve pacing.

### Confirmation of Isolation and the use of "Touch-ups"

After ablation, as with all PVI procedures, we confirm the presence of PV entrance and exit block with a circular mapping catheter. In the rare instances when PVI was not achieved with the cryoballoon alone, we have targeted the few remaining left atrial-PV connections with the Freezor Max 8-mm tip catheter (Medtronic, Minneapolis, MN). Although some centers aggressively pursue multiple cryoballoon applications until PVI is achieved, we prefer focal ablation "touch-ups" for PVs that prove initially difficult to isolate. Arguments for limiting the number of cryoballoon applications included the potential increased risk of phrenic nerve injury during right-sided ablation, and prolonged procedural time in the left atrium; both of which must be balanced against the increased costs of additional ablation catheters. It should be noted that the efficacy of PVI has not been shown to differ amongst the two approaches (>98% of patients achieve complete PVI with either a cryoballoon-exclusive or "hybrid" cryoballoon-cryofocal approach).[11] As such, it remains a matter of physician preference.

Other strategies proposed to enhance PVI and minimize reconnection include serial cryoapplications based on the "freeze-thaw-freeze" principal. Alternatively, some authors have advocated for the performance of sequential engagement and cryoablation using multiple different PV branches in order to create sequential overlapping lesions.

### End of the Procedure

At the end of the procedure, the presence of entrance block is reconfirmed in all PVs prior to removal of the transseptal sheath(s). While it has become standard practice to include a post-procedure observation period to monitor for acute reconnection after RF ablation, our sense is that such a waiting period is not necessary after CBA. In contrast to the relatively high rate of procedural reconnection observed in studies of RF-based AF ablation (up to 50% in some series), the rate of acute reconnection post CBA appears to be low. In studies employing waiting periods of up to 60 minutes, the pooled rate of acute recurrence among the 749 treated PVs was <1% [40,43,46-48]. Moreover, in contrast to RF-based ablation, the use of adjunctive pharmacologic agents such as adenosine and/or isoproterenol does not appear to provide additional prognostic information in patients undergoing CBA [40,49,50].

## Persistent AF

While the acute success rate for paroxysmal AF is high (>98%), cryoballoon-based PVI alone for patients with persistent AF has been associated with high rates of arrhythmia recurrence (45% 1-year freedom from recurrent AF).[11] Unlike patients with paroxysmal AF, catheter ablation of persistent and permanent AF may require more extensive ablation beyond PVI [51,52]. In this patient population, the left atrial substrate plays a prominent role in AF maintenance. Staged ablation strategies involving additional linear ablation and/or complex fractionated atrial electrogram-based (CFAE) ablation have yielded superior results [51,53]. Recently, a small hypothesis-generating study by Mansour et al. investigated an approach of cryoballoon PVI combined with conventional irrigated-RF based substrate modification in 22 participants with persistent AF.[54] The authors explored the utility of a step-wise approach beginning with cryoballoon-based PVI (22 participants) followed by CFAE ablation in those who had persistent or inducible AF (19 participants). Thereafter, linear ablation of the roof, mitral isthmus, and septum was performed if ablation did not terminate AF or if the arrhythmia changed to an atrial tachycardia or flutter (10 participants). After a mean follow-up of 6 months, the single procedure freedom from recurrent AF off AAD was a remarkable 86%. Based upon these results, the authors concluded that a combined cryoballoon and RF approach is feasible with favourable short-term maintenance of sinus rhythm. Prospective studies are required to determine whether the safety and efficacy of a hybrid cryoballoon approach merits adoption in clinical practice.

## Conclusion

Cryoballoon-based catheter ablation is a safe and effective technique for PVI with high acute and medium-term efficacy rates when used for paroxysmal AF. The rate of complications with CBA is relatively low, with the exception of a disproportionately increased incidence of phrenic nerve palsy (the majority of which are transient). Whether further benefits of cryoablation are realized will depend upon operator experience, refinements in the ablation technique, and evolutionary catheter development. Further studies, including direct comparison to conventional RF ablation are ongoing and will provide important insight into longer-term efficacy and safety.

## Figures and Tables

**Table 1:**
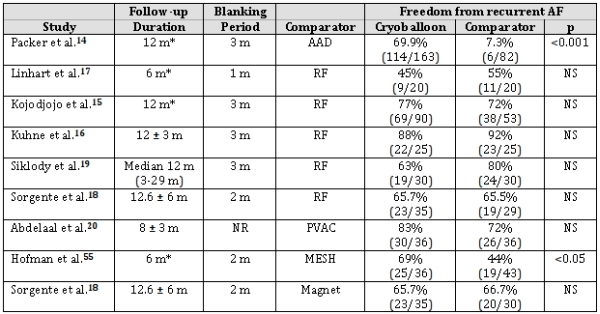
Studies Comparing Cryoballoon-Based Ablation To Other Rhythm Control Methods

AAD - Antiarrhythmic drugs; Magnet - Magnetic-assisted RF; MESH - Mesh Ablator catheter; NR - Not reported; NS - Not significant; PVAC - duty-cycled multi-electrode ablation; RF - convention radiofrequency catheter ablation
